# Calf deep veins are safe and feasible accesses for the endovascular treatment of acute lower extremity deep vein thrombosis

**DOI:** 10.1038/s41598-024-63782-6

**Published:** 2024-06-05

**Authors:** Xiande Zeng, Xixi Min, Wei Chen, Xiong Zeng, Zhinan Ju, Kanghui Dai, Weimin Zhou, Jiehua Qiu

**Affiliations:** Department of Vascular Surgery, The Second Affiliated Hospital of Nanchang University, Nanchang, 330006 Jiangxi China

**Keywords:** Deep vein thrombosis, Access, Calf deep vein, Endovascular treatment, Health care, Diseases

## Abstract

This study was designed to assess the optimal access route for the endovascular treatment of acute lower extremity deep vein thrombosis. This was a retrospective analysis of patients with acute lower extremity deep venous thrombosis who underwent endovascular treatment from February 2009 to December 2020. Patients underwent non-direct calf deep vein puncture (NDCDVP) from February 2009 to December 2011 and direct calf deep vein puncture (DCDVP) from January 2012 to December 2020. Catheter directed thrombolysis (CDT) was used to treat all patients in the NDCDVP group, whereas patients in the DCDVP group were treated with CDT or the AngioJet rhyolitic thrombectomy system. In patients exhibiting iliac vein compression syndrome, the iliac vein was dilated and implanted with a stent. Technical success rates and perioperative complication rates were compared between these two treatment groups. The NDCDVP group included 83 patients (40 males, 43 females) with a mean age of 55 ± 16 years, while the DCDVP group included 487 patients (231 males. 256 females) with a mean age of 56 ± 15 years. No significant differences were observed between these groups with respect to any analyzed clinical characteristics. The technical success rates in the NDCDVP and DCDVP groups were 96.4 and 98.2%, respectively (*P* > 0.05). In the NDCDVP group, the small saphenous vein (SSV)or great saphenous vein (GSV)were the most common access routes (77.1%, 64/83), whereas the anterior tibial vein (ATV) was the most common access route in the DCDVP group (78.0%, 380/487), followed by the posterior tibial vein (PTV) and peroneal vein (PV)(15.6% and 6.4%, respectively). Relative to the NDCDVP group, more patients in the DCDVP group underwent the removal of deep vein clots below the knee (7.2% [6/83] vs. 24.2% [118/487], *P* < 0.001). Moreover, relative to the NDCDVP group, significantly lower complication rates were evident in the DCDVP group (local infection: 10.8% vs. 0.4%, *P* < 0.001; local hematoma: 15.7% vs. 1.0%,* P* < 0.001). The position change rate was also significantly lower in the DCDVP group relative to the NDCDVP group (0% [0/487] vs. 60.2% [50/83], *P* < 0.001). The calf deep veins (CDVs) represent a feasible and safe access route for the endovascular treatment of lower extremity deep vein thrombosis.

## Introduction

Rates of venous thromboembolism (VTE), which included both pulmonary embolism (PE) and deep vein thrombosis (DVT) cases, affect 79–269 per 100,000 people, with rates varying among populations^1^. Lower extremity DVT (LEDVT) is a very serious condition that can cause high mortality rates during the acute stage followed by prolonged morbidity during the chronic stage^2^. A majority of PEs arise from LEDVTs, and cause mortality rates of 25% when untreated and 5–10% even with hospitalization^3^. Chronic complications that can develop following LEDVT are referred to as post-thrombotic syndrome (PTS), and can lead to the development of venous ulcers in ~ 15% of cases and venous claudication within 5 years in ~ 40% of cases, adversely impacting patient quality of life^4,5^. In order to minimize the risk of such complications, several different procedures have been designed to facilitate the rapid early removal of deep vein clots during the acute stage^6–9^.

The first thrombectomy procedure performed to treat acute LEDVT was reported in 1937 by Läwen, and this approach became increasingly popular through the 1950s–1960s^10^. However, such treatment can result in a range of vessel-related complications including vessel rupture, endothelial denudation, incisional infection, and vessel occlusion^11^. In order to avoid the open surgery-related complications, percutaneous approaches to thrombus removal have since been established and are widely used to treat acute LEDVT patients. Endovascular treatment strategies used in this setting include catheter-directed thrombolysis (CDT), pharmacochemical thrombectomy (PMT), and a combination of CDT and PMT^12,13^. The results of CaVenT trial (catheter-directed venous thrombolysis in acute iliofemoral veinthrombosis) showed that CDT reduced the occurrence of PTS to varying degrees at 2 and 5 years^14,15^. Although the acute venous thrombosis: thrombus removal with adjunctive catheter-directed thrombolysis^16^ (ATTARCT)trial reports that PMT does not reduce the risk of PTS, another study^17^ shows that CDT significantly reduces symptoms reduces PTS severity scores and significantly improves quality of life in venous patients.

The selection of an appropriate access route when performing the percutaneous treatment of LEDVT is critical, and the popliteal vein (PV) is the most commonly utilized access^14,18–21^, with the small and great saphenous veins (SSV or GSV) are optional access routes when performing CDT treatment^22^. All of these access routes are associated with the potential for certain complications, and there is thus a clear need for the establishment of a safe, feasible access that can maximize intraoperative patient comfort and achieve optimal perioperative outcomes. The present study was developed to assess whether the calf deep veins (CDVs: Anterior tibial vein, ATV; Posterior tibial vein, PTV; Peroneal vein, PNV) are safe and effective accesses when performing endovascular treatment in acute LEDVT patients.

## Patients and methods

### Study design

This was a retrospective study. All enrolled patients were recruited from the second affiliated hospital of Nanchang University from February 2009 to December 2020. Ethics Committee of the Second Affiliated Hospital of Nanchang University approved the study protocol, which was in accordance with Chinese law, the Declaration of Helsinki (2013), and TREND (Improving the reporting quality of nonrandomized evaluations of behavioral and public health interventions statement)^23^. Patients were separated into two groups, including the non-direct calf deep vein puncture (NDCDVP) group from February 2009 to December 2011 and the direct calf deep vein group puncture (DCDVP) group from January 2012 to December 2020. Informed consent for our study was obtained from all subjects and–or their legal guardians. Eligible patients were LEDVT patients that underwent endovascular treatment in the acute stage: Iliac vein, iliac-femoral vein, femoral-popliteal vein, or whole lower extremity deep vein thromosis. Patients were excluded if they: (1) only underwent inferior vena cava filter implantation, (2) only underwent inferior vena cava filter implantation and retrieval, or (3) had PEs that were treated via an endovascular approach.

### Interventions

**Figure 1:**
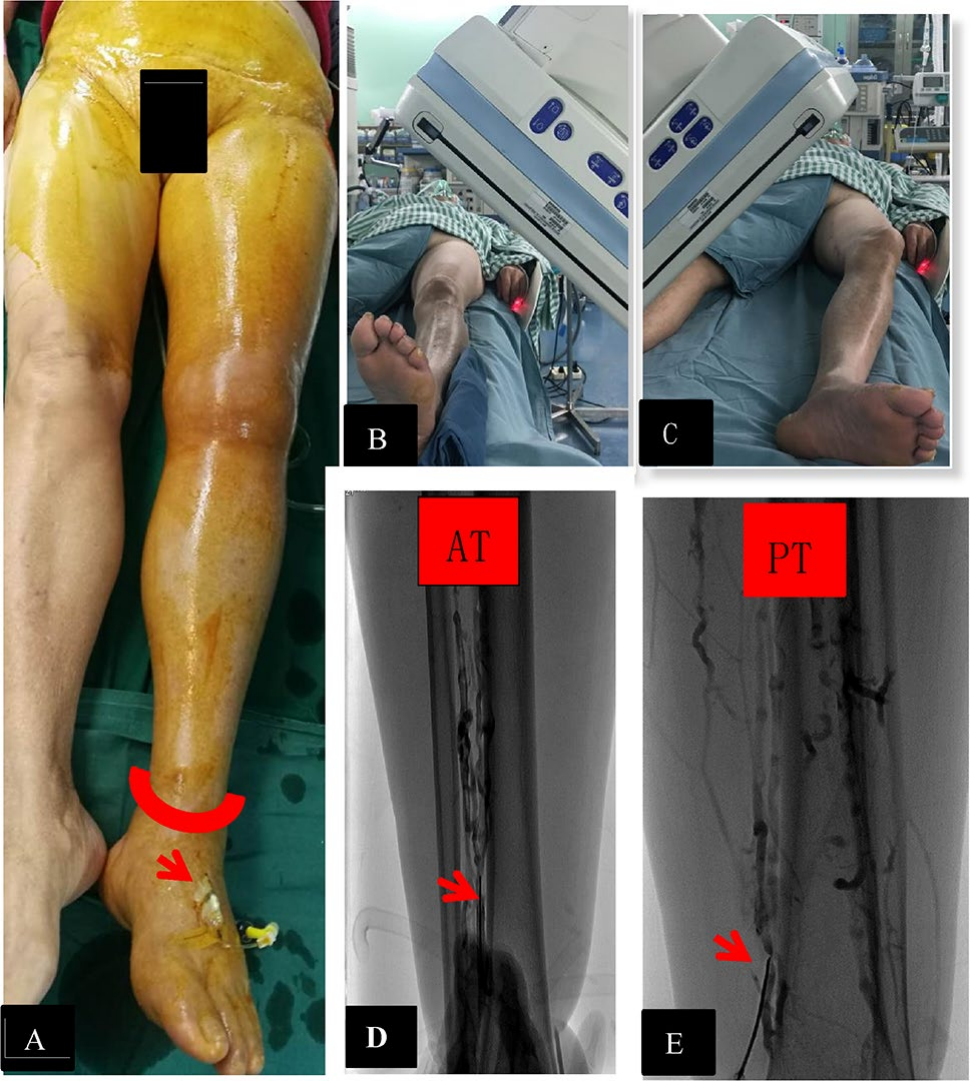
Overview of the calf deep vein puncture procedure. (**A**) The entirety of the affected limb and the contralateral inguinal region were sterilized, and an indwelling needle was inserted into the dorsal vein of the foot with the elastic bandage on the ankle (Arrow and Semicircle). (**B**) The position of the DSA guidance for anterior tibial vein and peroneal vein puncture. (**C**). The position of the DSA guidance for posterior tibial vein puncture. (**D**, **E**) Puncture of the anterior and posterior veins was performed under real-time X-ray guidance using an appropriate contrast agent.

A team of vascular surgeons from the Second Affiliated Hospital of Nanchang University performed all procedures in this study. All patients underwent routine examination (includes routine blood, biochemistry (liver and kidney function, electrolytes, etc.), coagulation function, blood transfusion four (syphilis antibody, HIV antibody, antibody to hepatitis C virus, antibody to hepatitis E virus) stool routine, urine routine, electrocardiography, chest X-ray, cardiac ultrasound and so on) upon admission, and the diagnosis of LEDVT was confirmed based on color ultrasound(EDAN portable fully digital diagnostic colour ultrasound system U60, probe model L742UB), serum D-dimer, and venography results. After completing the appropriate consent forms, patients were transferred to the interventional operating room. First, ascending venography using a dorsal foot vein was conducted to confirm the extent of thrombus involvement. Inferior vena cava (IVC) venography was performed from a contralateral femoral vein (FV) access to assess whether thrombi were present in the IVC. An IVC filter (Cordis OptEase, Cook Celect, or Lifetech Aegisy) was then implanted and expanded ~ 1 cm below the renal vein. In the NDCDVP group, access for the CDT catheter, balloon, and stent was achieved via the SSV or GSV via an incision or puncture, or via the popliteal vein (PV) via ultrasound-guided puncture. In the DCDVP group, contrast agent was injected through an indwelling needle in the dorsal vein of the foot with an elastic bandage around the ankle (Fig. 1). Then, a metal needle was used to puncture the ipsilateral anterior or posterior tibial vein (ATV or PTV) or peroneal vein (PNV) under DSA fluoroscopy (real-time X-ray with contrast agent, in low energy X-ray exposure mode) guidance (Fig. 1), and an introducer sheath was delivered into the punctured vein with a guide wire. Then, 0.035 inch, 150 cm Loach Guide Wire was placed and used to gently guide a 4F single curved catheter to the iliac vein and IVC, as confirmed via venography. The wire was then exchanged for a 0.035 inch, 260 or 300 cm wire to establish the working pathway, after which the CDT catheter, AngioJet rheolytic thrombectomy (ART) catheter (6F, 120 cm), balloon catheter, and stent were introduced through the wire into the thrombotic vein segment or iliac vein in an anterograde fashion. The iliac vein was then dilated and a stent was implanted (Boston, Wallstent; Bard, Luminexx; ev3, Protégé GPS) in cases of iliac vein compression syndrome that was confirmed following thrombus removal. If the Wallstent diameter was > 14 mm, additional access from the ipsilateral FV was required. And the filter was taken out when the CDT treatment was finished. For PMT treatment patients, the filter was removed after PMT treatment finished.

The primary outcome of this study was the technical success rate, corresponding to the rate that access for appropriate endovascular treatment materials (catheter and balloon) could be achieved. Secondary outcomes included the time of the access puncture, rates of positional change during the procedure (Changing patient position during imaging and treatment), whether DVTs located below the knee were removed, whether an artery was mistakenly punctured, and whether local infections or hematomas developed within 1 month after the procedure.

### Statistical analysis

SPSS v 17.0 (SPSS Inc., IL, USA) was used for all statistical analyses. Continuousdata are given as means ± SD, while categorical data are given as frequencies and percentages. Differences were compared between groups using t-tests and chi-square tests for quantitative and qualitative data, respectively, with *P* < 0.05 as the significance threshold.

## Results

In total, 570 patients were included in this study. The NDCDVP group enrolled 83 patients (40 male, 43 female with a mean age of 55 ± 16 years, while the DCDVP group enrolled 487 patients (231 male, 256 female) with a mean age of 56 ± 15 years. For details regarding clinical characteristics in these patients such as the affected limb and other factors, see Table 1. There were no significant differences in any of the analyzed characteristics between these two patient groups (Table 1).

**Table 1:** Clinic Characteristics of patients.

	UDCDVP	DCDVP	*P*-Value
Period	02.2009-12. 2011	01.2012-12. 2020	–
Total patients	83	487	–
Age (year)	55 ± 16	56 ± 15	0.7380
Gender(M–F)	40 (48.2%)	231 (47.4%)	0.8981
	43 (51.8%)	256 (52.6%)	
Operation related	29	167	0.9085
Affected limb (R–L)	24 (28.9%)	146 (30.0%)	0.8448
	59 (71.1)	341 (70.0%)	
Involved range			
Iliac vein	25 (30.1%)	148 (30.4%)	0.9606
Iliac-femoral vein	9 (10.8%)	72 (14.8%)	0.3416
Femoral-popliteal vein	10 (12.0%)	51 (10.5%)	0.6677
Whole veins	39 (47.0%)	216 (44.4%)	0.6554

NDCDVP: non-directly calf deep vein puncture group;

DCDVP: directly calf deep vein group puncture group;

Whole veins: The veins from iliac vein to the calf deep veins. M, Male; F, Female; R, Right; L, Lift.

**Figure 2:**
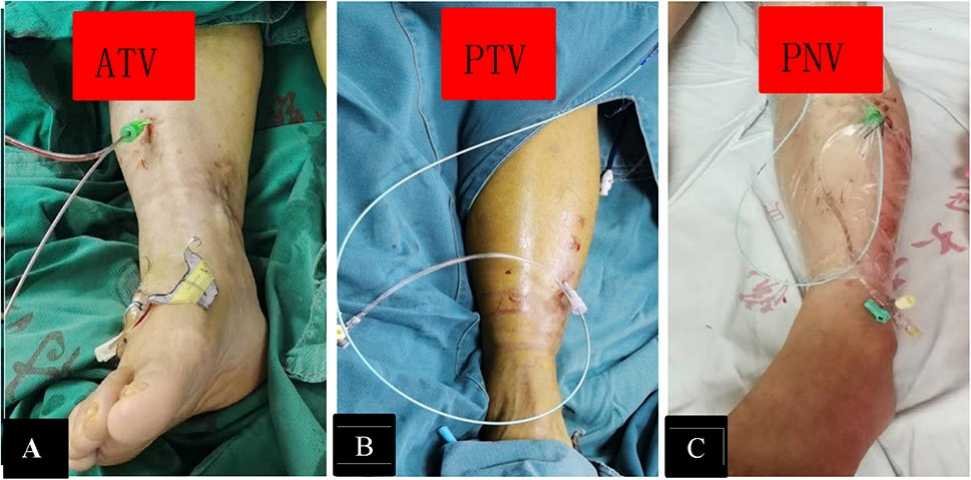
Representative examples of the use of each calf deep vein as an access route. (**A**) The anterior tibial vein. (**B**) The posterior tibial vein, and C. The peroneal vein was used as an access route for the endovascular treatment of LEDVT.

**Figure 3:**
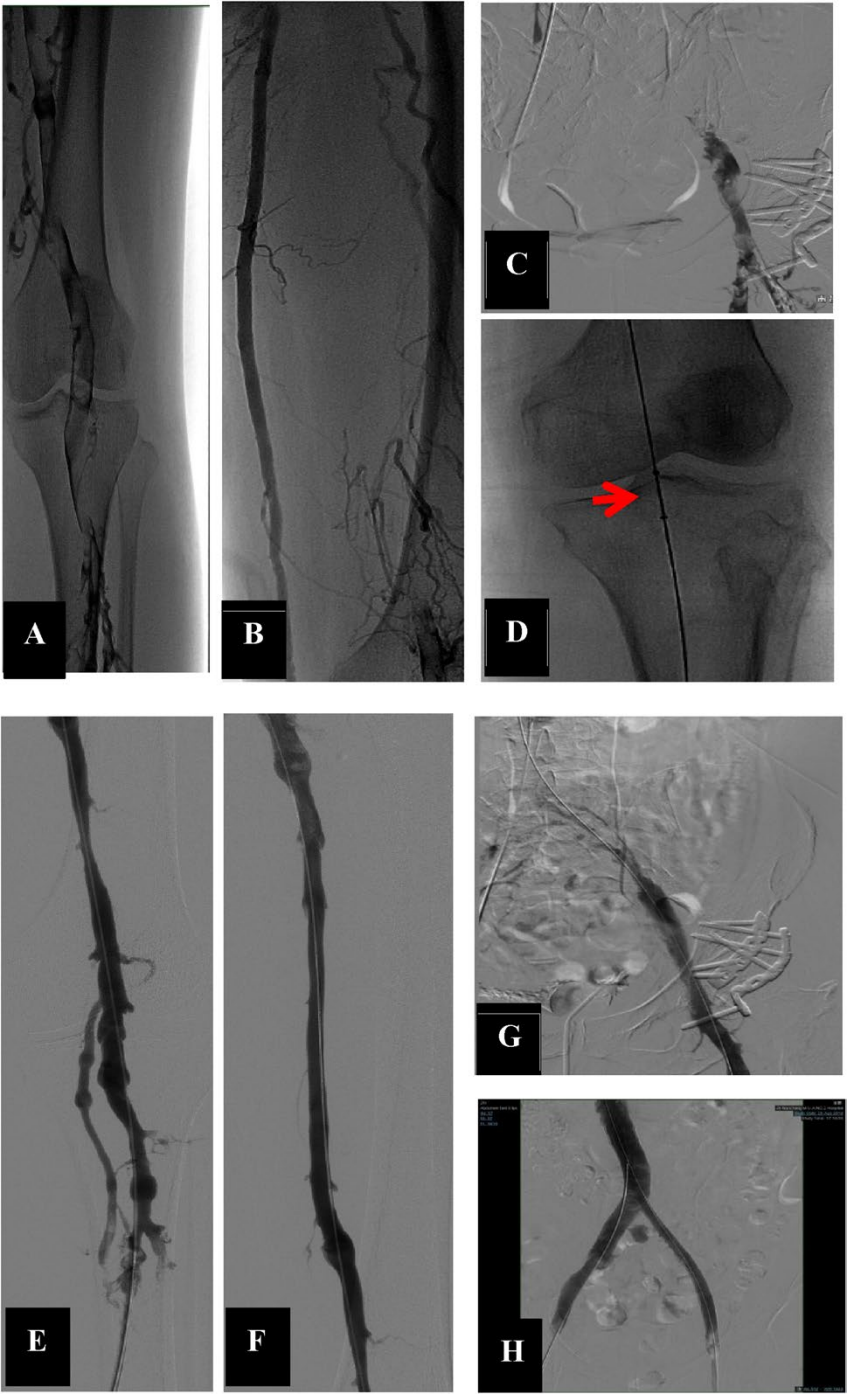
One patient exhibiting LEDVT and iliac vein compression syndrome underwent treatment via AngioJet rheolytic thrombectomy and iliac vein stent implantation. (**A**–**C**) The complete LEDVT in this patient. (**D**) The AngioJet rheolytic thrombectomy catheter. (**E**–**G**). The immediate effects following pharmacochemical thrombectomy. (**H**) The immediate effects on the iliac vein following stent implantation.

The technical success rates in the NDCDVP and DCDVP groups were 96.4% and 98.2%, respectively (*P* > 0.05). The position change rate in the NDCDVP group was significantly higher than in the DCDVP group (60.2% [50/83] vs. 0% [0/487], *P* < 0.001). The largest introducer sheath in the NDCDVP group was a 10F sheath, while the largest in the DCDVP group (for CDV access) was an 8F sheath, and introducer sheath details are provided in Table 2. The SSV or GSV was the most common access site in the NDCDVP group (77.1%, 64/83), and access was achieved via incision in 39 patients. PV access was achieved by puncture in the other patients, and endovascular treatment was discontinued for 3 patients due to a failure to obtain access. The ATV was the most common access in the DCDVP group (78.0%, 380/487), followed by the PTV and PNV at rates of 15.6% and 6.4%, respectively (Table 2, Figs. 1 and 2). Access via the CDV failed in 9 patients (Table 2), and PV access was instead used in these cases. Moreover, additional ipsilateral FV access was required in 104 patients for a large-diameter iliac vein stent (Boston, Wallstent). Significantly higher rates of below-the-knee DVT removal were observed in the DCDVP group relative to the NDCDVP group (24.2% [118/487] vs. 7.2% [6/83], *P* < 0.001). A representative total DVT patient in the DCDVP group is shown in Fig. 3. Overall, 51 and 310 patients underwent iliac vein stent placement in the NDCDVP and DCDVP groups, respectively. Artery mis-puncture rates were lower in the DCDVP group relative to the NDCVP group, but the difference was not significant (0.6% [3/487] vs. 2.4% [2/83], *P* = 0.1053). Rates of local infection and hematoma also differed significantly between these groups (infection: 10.8% in NDCDVP group vs. 0.4% in DCDVP group, *P* < 0.001; hematoma: 15.7% in NDCDVP group vs. 1.0% in DCDVP group,* P* < 0.001) (Table 2).

**Table 2:** The comparison of outcomes between two groups.

	NDCDVP	DCDVP	*P*-Value
Success rate of AO	80–83	478–487	0.2965
Change position rate	50 (60.2%)	0 (0%)	< 0.001
TAO	16.1 ± 6.4	5.4 ± 1.2	< 0.001
The sheath size			
5 F	29 (34.9%)	115 (23.6%)	–
6 F	10 (12.0%)	326 (66.9%)	–
8 F	20 (24.1%)	46 (9.4%)	–
10F	24 (28.9%)	0 (0%)	–
Access site			
Incision	41 (49.4%)	0 (0%)	
Puncture	42 (50.6%)	487 (100%)	
SSV or GSV	64 (2 Failed) (77.1%)	0 (0%)	–
PV	19 (1 Failed) (22.9%)	9 (1.85)	–
ATV	0 (0%)	315 (3Failed) (64.7%)	–
ATV + I-FV	0 (0%)	65 (13.3%)	–
PTV	0 (0%)	48 (2 Failed) (9.9%)	–
PTV + I-FV	0 (0%)	28 (5.7%)	–
PNV	0 (0%)	20(4 Failed) (41.95)	–
PNV + I-FV	0 (0%)	11 (2.3%)	–
BTDV clot treated	6 (7.2%)	118 (24.2%)	< 0.001
Artery mis-puncture	2 (2.4%)	3 (0.6%)	0.1053
Iliac vein stent	51 (61.4%)	310 (63.7%)	0.8605
Local infection	9 (10.8%)	2 (0.4%)	< 0.001
Local hematoma	13 (15.7%)	5 (1.0%)	< 0.001
Numb of calf skin	12 (14.5%)	2 (0.4%)	< 0.001

AO, Access obtained; TAO, Time of access obtained; F, French; SSV, Small saphenous vein; GSV, Great saphenous vein; PV, Popliteal vein; ATV, Anterior tibial vein; PTV, Posterior tibial vein; PNV, Peroneal vein; I, Ipsilateral; BTDV, Below knee deep vein.

Change position: change position from prone to supine or the knee joint flexion.

## Discussion

Rapid thrombus removal is the first-line treatment for acute LEDVT^5,24,25^, as it can reduce the risk of complications at both the acute and chronic stages including PEs and PTS. Endovascular techniques have emerged as an advantageous approach to acute DVT treatment^19^, but the selection of an appropriate access route is essential to the success of this procedure. The results of the present study confirmed that the CDVs represent feasible and safe access routes for the endovascular treatment of DVT that can provide patients with a good level of perioperative comfort.

The use of the superficial calf veins as an access route for thrombolysis catheter placement in DVT was first described in 1996 by Cragg et al.^26^. The SSV has been frequently used as an access via puncture or incision^22^, and it has even been used as an access for thrombus suction devices in recent years^27^. However, the SSV exhibits unique characteristics including an anatomic relationship with the sural nerves^28,29^, contributing to potential procedural complications, particularly in patients in whom incision is performed. Numbness of the area innervated by damaged sural nerves is a relatively common complication that affects ~ 10% of patients^27^. The access route in most patients in the NDCDVP group in this study was the SSV, and about 13% of patients experienced numbness of the calf skin. The relative complications in our NDCDVP group was high. The reason may be large fraction of subjects suffered the adjunctive surgical incision for the access due to our skills inexpert in performing ultrasound and puncture veins. This rate was significantly lower in the DCDVP group, primarily as no incision was performed, thus avoiding damage to the sural nerves. Anatomical variations of the SSV are also common. Over 20% of SSVs do not empty into the PV, instead emptying into the GSV or femoral vein^27,28^. The SSV is thus not an effective access if clots have spread to the PV as it can be difficult for the thrombolysis catheter or thrombus suction devices to reach the thrombotic segment. Positional change is also often necessary for incisions made during the operation, and thrombolytic or anticoagulant drugs are administered during the treatment period, increasing rates of complications such as local bleeding, hematoma, and infection. Rates of these complications were low in the DCDVP group, potentially owing to the absence of any incision or position change and to the rest of care for the selected access sites.

The PV is also frequently used as an access route for the CDT and PMT treatment of DVT cases^30^. As this vein is located on the back of the body proximal to the popliteal artery and tibial nerve in the popliteal fossa, which is composed of loose connective tissue, ultrasound guidance is routinely needed to maximize the success of puncture via this route while minimizing complication rates. Even so, patients can experience complications including nerve injuries and pseudo-aneurysm^7,31^, and many need to turn from the prone to the supine position or knee joint flexion during this procedure, potentially causing discomfort while increasing the work for medical staff. This procedure can also be difficult for patients affected by fractures, paralysis, or who have just completed other operations. Recent surgery is a risk factor in 15–40% of DVT patients^31,32^, with 34% of patients in the present study having a history of recent surgery. In the DCDVP group, the need for patients to change position was obviated, in line with results of another prior study^33^. However, clots situated below the knee can be difficult to treat via this entry route.

Access via the contralateral femoral vein has also been reported^7^, but this procedure is associated with high failure rates. This may be due to the fact that the procedure is opposed to the femoral vein valve direction, and left-sided LEDVTs are more common than right-sided ones^31^. As many LEDVTs co-occur with iliac vein compression syndrome, this can make it challenging to advance devices through this venous region. In the present study, 89% of patients with left-sided LEDVTs also exhibited iliac vein compression syndrome. Access via this route may also injure the valve, increasing the risk of post-thrombotic syndrome^34^.

To overcome the above issues, CDVs were adopted as an access route for the endovascular treatment of DVT patients. Armon et al. first reported the use of PTV access for the CDT treatment of iliofemoral DVT in 1996, with further follow-up on this technique the following year^35^. More recently, Bendix et al. analyzed outcomes from 27 DVT patients treated via thrombolysis with access via the PTV, comparing this route PPV access and thereby confirming that the PTV is a safe and effective access option for treating cases of iliofemoral and femoropopliteal^33^. ATV access has also been described by Wang et al.^31,36^, who determined that this approach can be feasibly used for the safe and effective treatment of acute extensive LEDVT patients. Our institution began using CDV access as a standard approach when treating DVT patients beginning in 2012, with almost 500 patients having undergone treatment using this approach via the ATV, PTV, and PNV over the last decade. While the success rates in the NDCDVP and DCDVP groups in this study were similar, the rates of perioperative complications such as local infections and hematomas in the DCDVP group were significantly lower, in line with prior results reported by Bendix et al. who observed negligible post procedural local access site complications in their PTV access group^33^. There are six calf veins that can be used as an access route, necessitating the selection of the appropriate vein in a given patient. In the DCDVP group in the present study, CDV access failed in 9 cases, most often due to a history of CDV thrombosis. The diameter of the CDVs will increase when the proximal region is obstructed by a clot, increasing vascular pressure such that a combination of X-ray and contrast guidance can enable relatively easy access. No ultrasound guidance was used in this study^33^, with DSA guidance being used in all cases. DSA guidance offers advantages over ultrasound monitoring, allowing for the dynamic monitoring of the puncture route. During the process, the patients and interventional staff will suffer extra radiation. But the dosage is low. Because puncture is completed in a short period under the low energy X-ray exposure mode. Wang et al. reported several advantages associated with the ATV approach^36^, including the lack of any need to change positions during the operation, allowing for the effective lysis of thrombi located in deep veins below the knee while preventing mechanical injury to the vein valve and thereby better preserving valve function. As such, the appropriate selection of the ATV, PTV, or PNV is important, providing a better means of lysing thrombi located below the knee. However, there are some disadvantages to CDV access. For one, large sheath implantation can injure the CDVs, and the distance is too long to enable the delivery of a stent from the access to the iliac vein if an iliac vein stent is required. In this study, an 8F sheath was not associated with local site complications, and when a larger sheath was required we were able to overcome any issues by additionally utilizing the ipsilateral femoral vein following the dissolution of the target thrombi, but the complications were not increased even with the additional femoral vein approach. Moreover, 6F is sufficient for the use of stents produced by Bard or Ev3 Company. And with the improvement of technology, the prolife of stent may be become smaller, so this issue can overcome.

Several strategies can help improve the accuracy of CDV puncture, thereby minimizing the risk of multiple punctures or damage to nerves or arterial structures. We found venography to be necessary, with an appropriate contrast agent being injected via the indwelling needle in the dorsal vein of the foot while compressing superficial veins using an elastic bandage on the ankle (Fig. 1), thus allowing the confirmation of the extent of thrombus involvement and the selection of the most optimal CDV for use as an access route. The positioning of DSA guidance can then be modified to be parallel to the target vessel while avoiding the overlap of the target vessel and the bone in projections. A metal puncture needle represents the best choice for this procedure. The puncture direction must remain horizontal and parallel to the target vessel and vertical with the plate of DSA in the vertical direction, with the needle head being in the middle of the target vein in projections.

There are some limitations to this study. First, this was a single-center retrospective analysis, highlighting the need for future large-scale multi-center prospective analyses or controlled trials in order to more rigorously assess the value of CDVs as accesses when treating LEDVT. Second, the number of both group patients has big difference. Third, this study only assessed perioperative outcomes, while mid- and long-term results were not analyzed.

## Conclusions

These findings indicate that the CDVs are safe, feasible accesses that can maintain patient comfort during the endovascular treatment of LEDVT via both catheter-directed thrombolysis and pharmacochemical thrombectomy approaches.

## Data Availability

The datasets used and analyzed during the current study available from the corresponding author on reasonable request.
